# The impact of COVID-19 on self-management behaviours and healthcare access for people with inflammatory arthritis

**DOI:** 10.1186/s41927-021-00231-1

**Published:** 2021-10-18

**Authors:** Emma Caton, Hema Chaplin, Lewis Carpenter, Melissa Sweeney, Hsiu Yen Tung, Savia de Souza, James Galloway, Elena Nikiphorou, Sam Norton

**Affiliations:** 1 Health Psychology Section, 5th Floor, Bermondsey Wing, Guy’s Hospital, Institute of Psychiatry, Psychology and Neuroscience, King’s College London, London Bridge, London, SE1 9RT UK; 2 Centre for Rheumatic Diseases, King’s College London, London, UK

**Keywords:** COVID-19, Inflammatory arthritis, Qualitative, Self-management, Healthcare access

## Abstract

**Background:**

Inflammatory arthritis (IA) patients have been identified as at greater risk of severe illness from COVID-19. It is likely that lockdown restrictions (enforced by the UK government in response to the COVID-19 pandemic) and subsequent changes made to healthcare provision could impact patients’ abilities to effectively manage their condition. The aim of this study was to qualitatively explore the impact of COVID-19 on self-management behaviours and healthcare access for people with IA.

**Methods:**

Semi-structured interviews were conducted with 21 IA patients in June-July 2020, with nine follow-up interviews in November 2020. Interview schedules were developed with a Patient Research Partner and explored participants’ experiences of the COVID-19 pandemic. Interviews were conducted via telephone and analysed using inductive thematic analysis.

**Results:**

Participants were aged between 24 and 79 years (mean = 50.1, SD = 15.8), largely female (71%) and White British (86%). Four initial themes were identified: (1) Impact of COVID-19 on medication adherence, (2) Impact of COVID-19 on physical activity, (3) Impact of COVID-19 on diet, and (4) Impact of COVID-19 on healthcare access and delivery. Subthemes focused on positive and negative changes made to these areas, as well as behaviours which remained consistent. Follow-up interviews highlighted differences in participants’ experiences during the two lockdown periods.

**Conclusion:**

COVID-19 has affected patients’ abilities to manage their IA. Healthcare professionals need to recognise the ongoing impact of COVID-19 on patient self-management and healthcare access to ensure that adequate understanding and support is available to patients who may have inadequate disease control as a result.

**Supplementary Information:**

The online version contains supplementary material available at 10.1186/s41927-021-00231-1.

## Key messages

- The first COVID-19 lockdown had positive and negative impacts on physical activity, diet and medication-taking.
- The most common healthcare disruption was the introduction of telephone appointments to replace face-to-face consultations.
- Rheumatologists need to ensure they support patients who have inadequate disease control following the pandemic.

## Background

The UK government announced the first nationwide lockdown on 23rd March 2020; imposing strict restrictions on movement to prevent the spread of COVID-19 [1]. Inflammatory arthritis (IA) patients were identified as at greater risk of severe illness from COVID-19 and advised to follow strict ‘shielding’ guidelines. These guidelines encouraged patients to refrain from leaving their home, except for medical appointments, and to get food and medication delivered [2].

The unprecedented nature of the COVID-19 pandemic provided a unique opportunity to explore how people living with IA (e.g. rheumatoid arthritis, psoriatic arthritis, spondyloarthritis, connective tissue disease-related arthritis) are able to manage their condition whilst also observing lockdown restrictions. In order to reduce disease activity, IA patients are often prescribed immunosuppressant disease-modifying anti-rheumatic drugs (DMARDs) [3], which may lead to medication non-adherence in people with rheumatic disease [4] due to attributing higher risk of contracting COVID-19. Furthermore, the closure of all fitness facilities [5] and difficulties accessing food during the pandemic [6], have significantly altered the opportunities available to IA patients to engage in physical activity and healthy eating behaviours. The pandemic has also seen significant changes made to healthcare access and delivery as many face-to-face consultations were cancelled in favour of remote consultations [7].

It is likely that lockdown restrictions and subsequent changes made to healthcare provision, could impact the ability of IA patients to effectively manage their condition. The aim of this study was to qualitatively explore the impact of COVID-19 over the first two UK lockdown periods on self-management behaviours and healthcare access for people with IA.

## Methods

### Design and recruitment

This qualitative study was a sub-study nested within a larger longitudinal survey study (IA-COVID) exploring the long-term impact of COVID-19 on health-related quality of life for people with IA [8].

Participants were recruited via social media and relevant charity organisations. To be eligible for the study, participants needed to be 18+ years old, living in the UK and diagnosed with an IA condition. Participants were recruited to the qualitative sub-study following completion of the main survey, through additional research consent for contact.

Of the 338 participants from the main survey, 332 provided consent for further contact. People were approached in blocks of 10 until at least 20 had agreed to participate, including representation (i.e. at least 3 people) from each sex, ethnic minority groups, and the following IA condition groups: rheumatoid arthritis, psoriatic arthritis, spondyloarthritis, connective tissue disease-related arthritis. The study included patients who were taking conventional synthetic (cs) and/or biological (b) DMARDs, as well as those who had not been prescribed DMARDs for their condition. Sample size was determined a priori using a pragmatic approach; a sample of approximately 20 participants was deemed both feasible and appropriate given the scope of the study and the resources available to the research team for a medium thematic analysis project conducted as part of a Masters-level research programme [9].

Informed written consent was obtained from each participant at the beginning of both the survey and telephone interviews.

### Materials and procedure

Semi-structured interviews were conducted between 19th June and 22nd July 2020, with follow-up interviews conducted in November 2020. Interview schedules for both the baseline (see Additional File 1: Table S1) and follow-up interviews (see Additional File 2: Table S2) were developed by the researchers (EC, HC and SN) in collaboration with a Patient Research Partner (SdS).

The interview schedules aimed to explore participants’ early experiences of the COVID-19 pandemic. Questions were focused on participants’ mental and physical health during the lockdown period, clarity of the messages and guidance received, and impact of COVID-19 on their self-management behaviours and healthcare access.

The interview schedules were piloted with the Patient Research Partner (SdS) and modified appropriately. Patient interviews were conducted in English, via telephone, and lasted 15–30 min. The interviews were audio-recorded, with permission, and transcribed verbatim.

### Analysis

Data was analysed using inductive thematic analysis [10], which allowed for a deep, data-driven exploration into patients’ experiences. Transcripts were read and re-read before being systematically coded by the lead researcher (EC). Five transcripts were independently coded by another researcher (HC). Similar codes were collated and organised to form themes and subthemes. These were discussed within the research team to ensure that they were well-developed and representative of the data.

## Results

### Participants

Twenty-one participants took part in the baseline interviews in June and July 2020, (see Table 1). These participants were also invited to complete follow-up interviews in November 2020, nine of which agreed to take part. Participants were aged between 24 and 79 years old (mean = 50.1, SD = 15.8), were mostly female (71%) and White British (86%). Four IA conditions were reflected in the sample (Rheumatoid arthritis: *n* = 3; Psoriatic arthritis: *n* = 8; Spondyloarthritis: *n* = 7; Connective tissue disease-related arthritis: *n* = 3). Disease duration ranged from < 1 to 39 years (median = 7, IQR = 11.5). The majority of participants (*n* = 19) had been prescribed csDMARDs (21%), bDMARDs (31.6%), or a combination of both (47.4%) to help manage their IA.

**Table 1:** Characteristics of interview participants

Participant ID	Sex	Age	IA condition	Ethnicity	Geographical location
Participant 1*	Female	70	Rheumatoid arthritis	White British	West Midlands
Participant 2	Female	55	Rheumatoid arthritis	White British	Yorkshire and the Humber
Participant 3	Female	38	Rheumatoid arthritis	White British	London
Participant 4	Female	59	Psoriatic arthritis	White British	Yorkshire and the Humber
Participant 5	Female	48	Psoriatic arthritis	White (any other White background)	Glasgow
Participant 6	Female	39	Psoriatic arthritis	White British	South East Wales
Participant 7*	Female	35	Psoriatic arthritis	White British	South East England
Participant 8*	Male	79	Psoriatic arthritis	White British	London
Participant 9*	Male	72	Psoriatic arthritis	White British	London
Participant 10*	Male	61	Psoriatic arthritis	White British	Yorkshire and the Humber
Participant 11*	Male	46	Psoriatic arthritis	White British	South East England
Participant 12	Female	67	Spondyloarthritis	White British	London
Participant 13	Female	59	Spondyloarthritis	White British	South East England
Participant 14	Female	43	Spondyloarthritis	White British	South East England
Participant 15*	Female	31	Spondyloarthritis	White British	London
Participant 16	Female	30	Spondyloarthrits	Mixed (any other Mixed background)	London
Participant 17*	Female	24	Spondyloarthritis	White British	East of England
Participant 18*	Male	31	Spondyloarthritis	White British	South West England
Participant 19	Female	68	Connective tissue disease- related arthritis	Black or Black British	West Midlands
Participant 20	Female	48	Connective tissue disease-related arthritis	White British	South East England
Participant 21	Male	49	Connective tissue disease-related arthritis	White British	West Midlands

*Participants who took part in the follow-up interviews in November 2020

### Themes

Four main themes were generated from the data: (1) *Impact of COVID-19 on medication adherence,* (2) *Impact of COVID-19 on physical activity,* (3) I*mpact of COVID-19 on diet* and (4) *Impact of COVID-19 on healthcare access and delivery.* A full summary of themes and subthemes can be found in Table 2.

**Table 2:** Summary of themes and subthemes

Themes	Subthemes	Description
Impact of COVID-19 on medication adherence	Adherence to prescribed medication regimen	Most participants remained adherent to their prescribed medication regimens throughout the pandemic
	Lack of adequate information and reassurance	Some participants received very little advice about taking their medication during the pandemic and thus discontinued their medication regimen
Impact of COVID-19 on physical activity	Increased opportunity to engage in physical activity	For some participants, the COVID-19 lockdown provided the time and opportunity to engage in more physical activity and exercise
	Shielding guidelines restricting physical activity	Most participants expressed that the introduction of ‘shielding’ guidelines led to a decrease in their physical activity levels
	Seasonal changes restricting physical activity	Many participants expressed that they were unable to engage in outdoor physical activity during the second lockdown due to the poor weather
Impact of COVID-19 on diet	Increased capacity to plan healthy meals	Some participants expressed that their diet had improved during the two lockdown periods due to having the increased time and capacity to plan and prepare healthy meals
	Continuation of pre-pandemic dietary behaviours	Some participants did not make any changes to their diet during the COVID-19 pandemic
	Diet as emotional regulation	Most participants stated that their diet had got worse during the lockdown periods due to an increase in emotional eating
Impact of COVID-19 on healthcare access and delivery	Changes to healthcare access and delivery	All participants experienced changes to healthcare access during the initial stages of the COVID-19 pandemic, including the introduction of telephone appointments to replace face-to-face consultations
	Positive impact of changes to healthcare access and delivery	Many participants found the introduction of telephone appointments beneficial
	Negative impact of changes to healthcare access and delivery	Some participants stated that changes to healthcare access had had a negative impact on their health and wellbeing, especially if their disease was not well-controlled

### Theme 1: Impact of COVID-19 on medication adherence

During the first two COVID-19 lockdowns, most participants remained adherent to their prescribed medication regimens. However some participants discontinued their medication, with clearer information and reassurance required. Reassuringly, during the second lockdown period, fewer participants reported medication non-adherence.

#### Adherence to prescribed medication regimen

The majority of participants did not have any concerns about taking their medication during the pandemic and, as such, continued to take their prescribed medication as usual:

[Has anything changed in regard to taking your medication?] “No… I take my medication every three weeks.” (Participant 16, baseline)

Similarly, participants who experienced COVID-19 symptoms, or had been in close contact with someone who had, recognised that it was usually important to stop taking their immunosuppressants for a period of time. As a result, some participants paused their medication regimen in line with usual medication practices and advice:

I’d been in contact with someone with coronavirus and my nurse advised that I just isolate for two weeks anyway and not to take my injection for two weeks from that day. (Participant 17, baseline)

#### Lack of adequate information and reassurance

Some participants reported having stopped taking their medication, despite not having symptoms of, or contact with, COVID-19, and without consulting their healthcare team:

…probably slightly hysterical at the start like everyone else or some anxiety y’know about whether I should take it or not…just in case it made me more susceptible to um catch the virus (Participant 11, baseline)

This quote suggests that one of the main reasons for medication non-adherence was concerns from patients that their medication would make them more susceptible to contacting COVID-19. The term “slightly hysterical” provides an insight into the negative emotions experienced by patients upon having to decide whether to continue with their medication regimen.

Patients who did not have any concerns about taking their medication during the COVID-19 pandemic, noted that this was primarily due to the advice that they had received from their rheumatology team:

I didn’t really [have any concerns about taking medication] ‘cause my rheumatologist was very clear, um, when she saw me at my first consultation… (Participant 7, baseline)

This suggests that adequate information and reassurance from rheumatology services is important to reduce patients’ concerns about taking immunosuppressive medication during the pandemic. Even so, some participants felt that they did not receive adequate information regarding their medication and noted that they would have found it beneficial if more advice had been made available to them at the beginning of the pandemic:

I wish there had been advice at the start of the lockdown … I didn't really have any um advice concerning um my immunosuppressant drugs, you know, whether I should- what the best thing to do was. (Participant 11, follow-up)

### Theme 2: Impact of COVID-19 on physical activity

Physical activity (PA) was another behaviour that was influenced by the pandemic. The initial lockdown provided an opportunity for some IA patients to engage more in PA, whilst for others the restrictions led to a decrease in their exercise levels. Whilst some participants were able to increase their PA when lockdown restrictions eased in August and continue with these behaviours throughout the second lockdown, some reported a further decrease in PA during the second lockdown period due to seasonal changes in the weather.

#### Increased opportunity to engage in PA

For some participants, the COVID-19 lockdown had a positive impact on their PA levels:

…I've been consistently doing what I would call maybe moderate with occasional vigorous exercise five times a week which before [lockdown] it was like nothing... (Participant 6, baseline)

Encouragingly, data from the follow-up interviews suggests that many participants were able to maintain these positive exercise behaviours throughout the second lockdown period:

I haven't stopped the exercise that I took up in the first one [lockdown], so I’ve tried to get a walk in or run in every working day. (Participant 18, follow-up)

These increases in PA levels were directly attributed by participants to lockdown restrictions; the restrictions had lessened their work commitments therefore they had more time to exercise:

Because I weren’t at work, I was actually starting to exercise a little more. I started doing Joe Wicks every single day with my little boy. (Participant 17, baseline)

I am doing the things that I always wanted to do but never had the time in terms of exercise. (Participant 15, baseline)

These quotes also demonstrate other positive outcomes from initial lockdown such as ability to spend more time with family. Additionally, lockdown restrictions gave people the opportunity to prioritise themselves over their workload and engage in activities that they have always wanted to do, which may also benefit their mental wellbeing in addition to their physical health.

#### Shielding guidelines restricting PA

Although some reported increased PA, the majority of participants noted that their PA levels decreased during the lockdown period:

I’m probably doing less physical activity at the moment overall. (Participant 21, baseline)

Participants suggested that the reduction in their PA resulted primarily from the government shielding guidelines:

…if we weren’t…in the middle of a pandemic and I wasn’t shielding, I’d definitely be going out for a walk a lot more. (Participant 7, baseline)

This shows that, for individuals who usually exercise by walking or participating in other PA outside, the government restrictions greatly impaired their ability to engage in these behaviours and caused their PA levels to reduce.

#### Seasonal changes restricting PA

Environmental factors were also identified as a barrier to PA during the COVID-19 pandemic. For example, during the second lockdown period, some participants who had previously increased their PA noted that they were now unable to engage in outdoor exercise due to the poor weather:

this second lockdown the weather has been awful generally, so doing exercise has been a little bit more of an effort…I'm not doing nearly as much exercise in this second lockdown as I did in the first. (Participant 10, follow-up)

### Theme 3: Impact of COVID-19 on diet

In relation to eating behaviours, some participants noted that their diet had improved as a result of having an increased capability to plan and prepare healthy food. Some participants were able to maintain their pre-pandemic dietary behaviours whilst other noted that their diet had got worse due to the emotional challenges of lockdown. For the majority of participants, dietary behaviours remained consistent across the two lockdown periods.

#### Increased capacity to plan healthy meals

Some participants expressed that their diet had improved during the first two lockdown periods due to having more time to plan and prepare healthy meals.

I’m, um, shopping better. I’m planning meals, um, which is making me eat healthier. (Participant 14, baseline)

I have more time to think about healthy options… more time to think about what I'm eating and how I'm cooking. (Participant 15, follow-up)

Changes to food access due to difficulties going to the supermarket or accessing delivery slots also led to improvements in diet, as participants were encouraged to shop more conscientiously:

…because I wasn’t going to the shops… I found a fruit and veg supplier…I got these amazing fruit and veg boxes. (Participant 15, baseline)

#### Continuation of pre-pandemic dietary behaviours

Several participants did not experience any changes to their diet during the pandemic.

We eat reasonably healthy…I think our diet’s pretty much the same. (Participant 2, baseline)

This suggests that those who were already engaging in healthy eating behaviours pre-pandemic were able to maintain this throughout the lockdown periods.

#### Diet as emotional regulation

Most participants stated that their diet had been worse during the first lockdown period, especially in relation to the type and amount of food that they were consuming:

We were eating probably food that we wouldn't normally eat; we were a little less strict… we were comfort eating. (Participant 13, baseline)

The term ‘comfort eating’ implies that participants may be using food to regulate their emotional responses to COVID-19. Indeed, as the pandemic continued into a second lockdown, ‘comfort eating’ behaviours also continued, causing some participants to gain weight:

I mean I've been putting weight on, I have put about a stone on across lockdown, and I mean that's entirely due really to comfort eating and boredom. (Participant 1, follow-up)

### Theme 4: Impact of COVID-19 on healthcare access and delivery

The COVID-19 pandemic saw several changes being made to how healthcare services, such as rheumatology appointments and blood tests, were delivered. These changes were perceived both positively and negatively.

#### Changes to healthcare access and delivery

The most commonly reported change was the introduction of telephone appointments to replace face-to-face consultations. Remote approaches were applied to most healthcare services, including both rheumatology and GP consultations:

…various hospital appointments, um, with the consultants were over the phone rather than me going to the hospital… (Participant 8, baseline)

Secondary care (e.g. ophthalmology, podiatry) and blood test services were also disrupted during the pandemic, with many appointments being cancelled or postponed until after the lockdown period:

I can't reach to cut my own toenails, um, so that obviously going to, you know, the foot clinic in the [local hospital] has all been cancelled. (Participant 12, baseline)

#### Positive impact of changes to healthcare access and delivery

Some participants found the healthcare changes to be very beneficial. For example, Participant 6 noted that during their telephone consultations, they perceived healthcare professionals to be more receptive to their concerns:

…since lockdown they've been a lot more attentive and give me more time and listened a bit more to whatever it is I'm going through. (Participant 6, baseline)

It was also expressed that the use of telephone consultations may have economic benefits longer term for the healthcare system as well as saving time for both the service and patients:

I think it's a very good idea to, um, to do this [telephone appointments]… it must save a lot of time and money and also it saves a lot of, um, time from the patient’s perspective. (Participant 10, baseline)

There are a lot of issues that you can sort out over the phone… you do get things done quicker now. (Participant 17, follow-up)

#### Negative impact of changes to healthcare access and delivery

In some circumstances, the changes made to healthcare access had a negative impact on IA patients. For example, some participants reported experiencing increased health-related anxiety as a result of their blood monitoring being cancelled or postponed:

I’ve not had a blood test now in months so I don’t know how, y’know, my liver and my kidneys, um, are responding. I don’t know what my inflammatory levels are like…it would be nice to have confirmation, um, of that. (Participant 1, baseline)

Several participants also found the introduction of telephone appointments to replace face-to-face consultations unhelpful:

I don't think that just speaking to somebody on the phone you can, you know, they can really get to the gist of what's happening. I think they need to see me, um, and I didn't really find the telephone consultations helpful at all. (Participant 5, baseline)

Indeed, the lack of physical examination generated concerns for patients that decisions regarding their health were being made based on information from previous appointments:

…she obviously couldn’t see me, she couldn’t look at me she couldn’t feel my joints. She couldn’t, you know um, do anything apart from base her judgement on what she knew. (Participant 4, baseline)

Even though several IA patients found the telephone consultations to be unhelpful, they understood why changes to healthcare access had to be made during the pandemic:

…I completely understood why we had to do that…but just with the situation I was in [active disease] didn’t really help that much. (Participant 3, baseline)

The fact that Participant 3 found the telephone consultation unhelpful due to “the situation I was in” implies that effectiveness of telephone appointments may be linked to a patient’s disease activity. Indeed, Participant 2 expressed that they would have found the telephone appointments less challenging if their IA were more controlled:

I think it would be alright if I was quite stable [disease control] but because I’m not stable at the moment it’s quite frustrating. (Participant 2, baseline)

## Discussion

The purpose of this study was to explore the impact of COVID-19 on self-management and healthcare access for people with IA during the first two UK lockdown periods. Findings suggest that COVID-19 has had both positive and negative impacts on behaviours such as exercise, diet, and medication-taking, many of which remained consistent over-time. The analysis also helped identify some of the changes made to healthcare services during the pandemic and how these changes were received by IA patients. Based on these findings, recommendations to support IA patients during the COVID-19 pandemic have been suggested (see Table 3).

**Table 3:** Recommendations to support inflammatory arthritis (IA) patients during the COVID-19 pandemic

Recommendations
Continue to emphasise the importance of engaging in positive health behaviours (e.g. physical activity, diet, medication adherence) for the management of IA
Provide time-management advice to patients who want to prioritise engagement in positive health behaviours (e.g. engaging in physical activity, planning/preparing healthy meals)
Provide home-based exercise programmes to encourage patients to engage in physical activity at home
Signpost patients to mental health resources to help support patients who may be experiencing emotional challenges as a result of the pandemic
Encourage open discussions and provide reassurance to patients who may have concerns about taking their medication during the pandemic
Offer routine telephone appointments to patients who feel that their IA condition is well-controlled

In relation to PA, some participants reported that they were able to engage in more PA during the pandemic whilst others reported their PA levels had decreased. These changes in exercise behaviours were mostly attributed to the lockdown restrictions and shielding guidelines issued by the UK government. Data from our larger IA-COVID quantitative study [8] suggests that approximately half of IA patients reduced their PA levels during the two lockdown periods. Current research supports the notion that lockdown measures have led to a decrease in PA for clinically extremely vulnerable populations [11, 12]. In relation to rheumatic diseases, physical inactivity resulting from the COVID-19 pandemic may lead to worsened disease activity, increased symptoms of mental distress and a poorer overall quality of life [13]. Nevertheless, for some participants, being instructed to stay at home during the pandemic created the opportunity to engage in more PA because they were spending less time preoccupied with other commitments. Rheumatologists may want to emphasise the benefits of prioritising PA, especially home-based exercises, so that IA patients continue engage in these behaviours.

In addition, the UK lockdowns have also provided an opportunity for IA patients to engage in healthy eating behaviours; several participants noted that their diet had improved due to having more time to plan and prepare meals. Nonetheless, for most participants the lockdowns led to a worsening of diet. A cross-sectional survey study found that poor diet during the COVID-19 lockdown can be linked to a more negative mood [14]. In our study, several participants noted that they increased their consumption of unhealthy ‘comfort’ foods during the pandemic as a way of dealing with difficult emotions such as anxiety or boredom.

Regarding medication, the findings suggest that, for the majority of participants medication adherence was not influenced. Nevertheless, some participants did express discontinuing their medication due to concerns that it would make them more vulnerable to COVID-19. It is possible that these concerns may have been influenced by the high-levels of uncertainty regarding the use of DMARDs in the early stages of the pandemic; healthcare professionals were initially concerned that the use of immunosuppressive or immunomodulatory agents may predispose patients to severe COVID-19 infection [15]. Reassuringly, emerging literature suggests that the use of DMARDs is not associated with an increased risk of severe illness from COVID-19, and therefore do not need to be discontinued unless in the case of suspected or confirmed COVID-19 infection [16, 17]. In this study, adequate reassurance from healthcare professionals led to many participants continuing with their medication regimen during the pandemic. It is therefore necessary to ensure that the importance of medication adherence is more-widely communicated to IA populations to prevent the negative consequences of non-adherence.

Participants reported experiencing severe disruptions to healthcare services during the first lockdown period, most notably, the introduction of telephone appointments to replace face-to-face consultations [18]. Whilst some participants found the telephone appointments beneficial, several participants said that they found these consultations unhelpful because they were unable to be physically assessed. The findings imply that patient satisfaction with telephone appointments may be linked to disease activity, with patients with poor disease control finding the telephone appointments most ineffective. Healthcare services may want to consider offering routine telephone appointments as an option for patients who feel that their IA condition is well-controlled. Patient preference and perceived disease-activity should be taken into consideration when triaging for face-to-face or telephone consultations.

A limitation of this study is the lack of ethnic diversity within the sample. Only 14% of participants at baseline were from Black, Asian or Minority Ethnic (BAME) groups, which is not reflective of the ethnic diversity present in the UK. Given that BAME communities have been disproportionately affected by COVID-19 [19] and thus are likely to have different COVID-related experiences, findings from this study may not be representative of the wider population.

Furthermore, this study also lacks geographical diversity. In England, parts of the Midlands and Northern England (East Midlands, North West and North East) were not represented in the sample. Data from the Office for National Statistics suggests that COVID-19 infection rates varied across England, with North West England often experiencing higher infection rates, and thus greater lockdown restrictions, compared to other regions [20]. As such, the results from this study may not be reflective of the experiences of patients who reside outside the regions represented in this study.

Another limitation of the study is that only a relatively small number of rheumatoid arthritis (RA) patients were included in the sample, despite RA being the most common form of IA [21]. Due to the different disease experiences associated with each IA condition, the lack of RA patients in the study may impact the representativeness of the findings when considered in relation to the wider IA population. Nevertheless, the relatively large number of psoriatic arthritis (PsA) patients included in the sample is a strength of the study, as currently little research has been conducted exploring the experiences of PsA patients during the pandemic.

Findings from this study suggest that COVID-19 had a substantial impact on patients’ abilities to manage their IA condition. Future research would need to consider the longer-term impact of these behaviour changes on the health and wellbeing of IA patients and the impact this may have on public health services. Overall, healthcare professionals need to recognise the impact of COVID-19 on patient self-management and healthcare provision to ensure that adequate understanding and support in future is available to patients who may have inadequate disease control as a consequence.

## Supplementary Information

**Additional file 1. Table S1**: Baseline interview schedule.

**Additional file 2. Table S2**: Follow-up interview schedule.

## Data Availability

The data that support the findings of this study are available from the corresponding author, EC, upon reasonable request.

## Abbreviations

BAME: Black, Asian and Minority Ethnic
bDMARDs: Biological disease-modifying anti-rheumatic drugs
csDMARDs: Conventional synthetic disease-modifying anti-rheumatic drugs
COVID-19: Coronavirus disease 2019
DMARDs: Disease-modifying anti-rheumatic drugs
IA: Inflammatory arthritis
PA: Physical activity
PsA: Psoriatic arthritis
RA: Rheumatoid arthritis
