# Supplementary material for: The impact of COVID-19 on self-management behaviours and healthcare access for people with inflammatory arthritis
Source: BMC Rheumatol. 2021 Oct 18;5:58. doi: 10.1186/s41927-021-00231-1 (PMC8522124; doi:10.1186/s41927-021-00231-1)
Supplement: Supplementary file 1 — Additional file 1. Table S1: Baseline interview schedule. [file 41927_2021_231_MOESM1_ESM.docx]

**Additional File 1**

| Supplementary Table 1. Baseline interview schedule. |
| --- |
| *Baseline interview schedule* |
| Have you experienced any COVID-19 symptoms? [If no, move to Q2, if yes, ask prompt questions]  Prompts: What symptoms did you experience? Have you been tested for COVID-19, and if so what was the result? Did you feel the need to self-isolate and why? Have these symptoms had an impact on your arthritis, and if so, how? |
| Have you been able to self-isolate or socially distance yourself, and what does this mean for you?  Prompts: How have you been accessing your shopping and/or medication during this time? |
| Where has the information you have received regarding how to behave during this time come from?  Prompts: How clear have the messages or information you have received been? Have any messages been particularly helpful or unhelpful? |
| Could you tell me what the impact has been on your physical health during this time?  Prompts: How has your arthritis been? Have you experienced any other symptoms? |
| What have the disruptions to your usual inflammatory arthritis healthcare and treatments been during this time?  Prompts: Have you had to change your face-to-face consultations to telephone? Any changes to blood monitoring? Any difficulty contacting your rheumatology team? Any delays to referrals or operations? Do you have any concerns about taking or accessing your medications? How have you found adapting to these changes? |
| Could you tell me what the impact on your mental health has been during this time?  Prompts: How has it made you feel emotionally/in terms of your mood? |
| What changes have you made in how you look after yourself on a daily basis?  Prompts: How have your usual self-management behaviours like taking medication, exercise, diet or hygiene changed? Has anything stayed the same? |
| Is there anything else you would like to add about your experiences during this period? |
