# Supplementary material for: The impact of COVID-19 on self-management behaviours and healthcare access for people with inflammatory arthritis
Source: BMC Rheumatol. 2021 Oct 18;5:58. doi: 10.1186/s41927-021-00231-1 (PMC8522124; doi:10.1186/s41927-021-00231-1)
Supplement: Supplementary file 2 — Additional file 2. Table S2: Follow-up interview schedule. [file 41927_2021_231_MOESM2_ESM.docx]

**Additional File 2**

| Supplementary Table 2. Follow-up interview schedule. |
| --- |
| *Follow-up interview schedule* |
| Have you experienced any COVID-19 symptoms? [If no, move to Q2, if yes, ask prompt questions]  Prompts: What symptoms did you experience? Have you been tested for COVID-19, and if so what was the result? Did you feel the need to self-isolate and why? Have these symptoms had an impact on your arthritis, and if so, how? |
| Could you tell me what life has been like since we last spoke in June/July?  Prompts: What was life like once the first lockdown/self-isolation period was over? What has life been like since the second lockdown was implemented? |
| How has your physical health been during the second lockdown?  Prompts: How has your arthritis been? Have you experienced any other symptoms? |
| How has your mental health has been during the second lockdown?  Prompts: How has it made you feel emotionally/in terms of your mood? |
| Have you continued with any of the behaviour changes you made regarding how you look after yourself on a daily basis? Have you made any new changes?  Prompts: Have you continued/made any changes to your diet or exercise behaviours? Are you still socially distancing or practicing increase hygiene measures e.g. regular handwashing, sterilising contact surfaces? Do you wear a face mask when you are going to be in a crowded or indoor space? Do you keep in contact with family/friends? |
| Have you experienced any ongoing or new disruptions to your usual IA healthcare and treatments?  Prompts: Have you have to continue consultations by telephone? What did you like/dislike about the telephone consultations? Any issues about taking or accessing your medications? How have you found adapting to these changes? |
| Do you have any concerns for the future following this period? |
| Do you think you will have a COVID-19 vaccine when it becomes available? |
| Do you have any advice for how messages to people with IA conditions (either from the government or your healthcare team) could be delivered more clearly in the future if a similar situation were to happen again? |
| Is there anything else you would like to add about your experiences during this period? |
